# Elastic and Muscular Arteries Differ in Structure, Basal NO Production and Voltage-Gated Ca^2+^-Channels

**DOI:** 10.3389/fphys.2015.00375

**Published:** 2015-12-15

**Authors:** Arthur J. A. Leloup, Cor E. Van Hove, Annick Heykers, Dorien M. Schrijvers, Guido R. Y. De Meyer, Paul Fransen

**Affiliations:** ^1^Laboratory of Physiopharmacology, Department of Pharmaceutical Sciences, University of AntwerpAntwerp, Belgium; ^2^Laboratory of Pharmacology, Faculty of Medicine and Health Sciences, University of AntwerpAntwerp, Belgium

**Keywords:** basal nitric oxide, voltage-gated calcium channels, elastic arteries, muscular arteries, arterial compliance, arterial stiffness, diltiazem

## Abstract

In the last decades, the search for mechanisms underlying progressive arterial stiffening and for interventions to avoid or reverse this process has gained much attention. In general, arterial stiffening displays regional variation and is, for example, during aging more prominent in elastic than in muscular arteries. We hypothesize that besides passive also active regulators of arterial compliance [i.e., endothelial and vascular smooth muscle cell (VSMC) function] differ between these arteries. Hence, it is conceivable that these vessel types will display different time frames of stiffening. To investigate this hypothesis segments of muscular arteries such as femoral and mesenteric arteries and elastic arteries such as the aorta and carotid artery were isolated from female C57Bl6 mice (5–6 months of age, *n* = 8). Both microscopy and passive stretching of the segments in a myograph confirmed that passive mechanical properties (elastin, collagen) of elastic and muscular arteries were significantly different. Endothelial function, more specifically basal nitric oxide (NO) efficacy, and VSMC function, more specifically L-type voltage-gated Ca^2+^ channel (VGCC)-mediated contractions, were determined by α_1_-adrenoceptor stimulation with phenylephrine (PE) and by gradual depolarization with elevated extracellular K^+^ in the absence and presence of eNOS inhibition with L-NAME. PE-mediated isometric contractions significantly increased after inhibition of NO release with L-NAME in elastic, but not in muscular vessel segments. This high basal eNOS activity in elastic vessels was also responsible for shifts of K^+^ concentration-contraction curves to higher external K^+^. VGCC-mediated contractions were similarly affected by depolarization with elevated K^+^ in muscular artery segments or in elastic artery segments in the absence of basal NO. However, K^+^-induced contractions were inhibited by the VGCC blocker diltiazem with significantly higher sensitivity in the muscular arteries, suggestive of different populations of VGCC isoforms in both vessel types. The results from the present study demonstrate that, besides passive arterial wall components, also active functional components contribute to the heterogeneity of arterial compliance along the vascular tree. This crucially facilitates the search for (patho) physiological mechanisms and potential therapeutic targets to treat or reverse large artery stiffening as occurring in aging-induced arterial stiffening.

## Introduction

Ventricular pressure waves travel along the arterial tree from high-conductance, elastic arteries to high-resistance muscular arterioles. Here, wave reflection leads to summation of forward- and backward-traveling pressure waves with systolic blood pressure increasing up to 14 mm Hg between aortic root and brachial artery (Safar, 2010). It is obvious that functional and morphological properties of elastic and muscular arteries differ considerably. Elastic arteries, such as the aorta and the carotid artery, contain more elastin per unit area and have important pulse-smoothing properties of the pressure wave originating in the left ventricle. On the other hand, the muscular arteries such as femoral or mesenteric arteries have a relatively higher smooth muscle to elastin content, distribute the blood according to moment-to-moment needs and are more capable of vasoconstriction and dilation.

Progressive large artery stiffening with aging is the predominant cause of increased pulse pressure, a marker of cardiovascular risk in the general population (Benetos et al., 1993) and a predictor of cardiovascular events (Mitchell et al., 1997). Some studies have demonstrated that arterial stiffness increases progressively with age only in the elastic arteries, but not in muscular arteries (Laurent et al., 1994; Ruitenbeek et al., 2008; Borlotti et al., 2012; Zhang et al., 2013). To date, this interesting discrepancy is mainly attributed to the observation that age-associated geometrical changes are not homogenous along the arterial tree (Benetos et al., 1993) and that elastin fragmentation occurs predominantly in the elastic arteries, where the stretch amplitude is high (O'Rourke and Hashimoto, 2007). A study that compared geometrical and functional (i.e., stiffness) parameters in the carotid and radial arteries of young and elderly subjects reported that, during aging, both vessel types undergo structural remodeling (increased internal diameter and intima-media thickness) while stiffening occurs only in the elastic arteries (Bortolotto et al., 1999). This observation points to different regulations of arterial compliance in different vessel types and a complex interplay between large and small arteries in the development of arterial stiffness and hypertension (Laurent et al., 2009).

In the recent years, the evidence is growing that not only passive components determine arterial compliance. Indeed, intrinsic vascular smooth muscle cell (VSMC) stiffness and active vessel wall components (i.e., NO bioavailability and VSMC tonus) affect arterial compliance as well (Sehgel et al., 2013). An important active vessel wall component that regulates arterial stiffness, especially with respect to aging, is endothelium-derived nitric oxide (NO; Safar et al., 2001; Fitch et al., 2006; Bellien et al., 2010; Vayssettes-Courchay et al., 2011; Isabelle et al., 2012). Moreover, VSMC function shows age-dependent alterations. Indeed, in both normotensive and hypertensive rats, voltage-gated Ca^2+^ channel (VGCC) expression and the therapeutic potential of VGCC blockers decreased with age (Fukuda et al., 2014). Although the clinical interest in arterial stiffness as an independent predictor of cardiovascular complications (Laurent et al., 2006), has grown in the last decades, the fundamental characteristics of active regulators of arterial compliance (i.e., basal NO efficacy and VGCC-mediated contractions) in different vessel types have never been reported.

In the present study, we investigated whether basal NO activity and VGCC-mediated contractions differed between the smaller, muscular arteries (femoral and mesenteric arteries) and the larger, elastic conduit vessels (aorta and carotid artery) of C57Bl6 mice. To the best of our knowledge this is the first study to report differences in basal NO production and VGCC-mediated contraction curves in elastic and muscular mouse arteries. We speculate that the different physiological behavior of elastic and muscular arteries at young (adult) age are linked to the well-known observation that arterial stiffness develops differently with aging.

## Materials and methods

### Animals

The studies were approved by the Ethical Committee of the University of Antwerp, and all experiments were performed conform to the Guide for the Care and Use of Laboratory Animals published by the US National Institutes of Health (NIH Publication No. 85–23, revised 1996). Female C57Bl6 mice (*n* = 8, food and water *ad libitum*, 12/12 light-dark cycle) were used at the age of 5–6 months.

### Preparation of the arterial segments

Mice were sacrificed by perforating the diaphragm under anesthesia (sodium pentobarbital, 75 mg kg^−1^, i.p.). The thoracic aorta, carotid, femoral, and first order mesenteric artery were dissected systematically and stripped of adherent tissue. Vessel segments (width < 2 mm) were mounted in a four-channel wire myograph (DMT, Denmark) and immersed in Krebs Ringer solution (37°C, 95% O_2_/5% CO_2_, pH 7.4) with (in mM): NaCl 118, KCl 4.7, CaCl_2_ 2.5, KH_2_PO_4_ 1.2, MgSO_4_ 1.2, NaHCO_3_ 25, CaEDTA 0.025, and glucose 11.1. When possible, aortic, femoral, carotid and first order mesenteric artery segments of one mouse were mounted in parallel. In total, 4 aortic, 6 femoral, 8 carotid, and 8 mesenteric segments were investigated.

After a short equilibration period of 30 min, the segments were gradually stretched (200, 100, 50, or 25 μm increments) from 0 mN/mm to tensions according stresses above 13.3 kPa (100 mm Hg). After this passive stretch protocol, the segments were set at the internal circumference according to the 13.3 kPa stress (normalization factor = 0.9; Slezak et al., 2010). The internal circumference of the different arteries was calculated as [(2^*^Δμm stretch) + (4^*^r) + (2^*^r^*^Π)] with r, the radius of the wire (20 μm). Then, transducers were re-set to zero tension in order to measure active tension upon addition of 50 mM K^+^ or 10 μM phenylephrine (PE). High K^+^—solutions were prepared by replacing NaCl with equimolar KCl. Contractile tension was measured and reported in mN/mm (Van Hove et al., 2009).

### Vascular reactivity

Basal NO activity can be determined with high accuracy and sensitivity by measuring the inhibitory action on contractions induced by α_1_-adrenoceptor stimulation with PE and by measuring shifts of the depolarization-mediated window contraction curves (Fransen et al., 2012a,b; van Langen et al., 2012, 2013). Therefore, contractions by PE and shifts of window contraction curves were measured before and after inhibition of basal NO formation by endothelial NO synthase (eNOS) with 300 μM *N*^Ω^-nitro-L-arginine methyl ester (L-NAME). Window contraction curves were constructed from K^+^ concentration-response curves. The extracellular K^+^ was gradually increased by isosmotic replacement of Na^+^ for K^+^ and the segments were stepwise clamped to more depolarized membrane potentials. The first derivative of the concentration-contraction curves revealed the window contraction curves, which correlate well with window L-type Ca^2+^ influx as measured in isolated VSMC (Fransen et al., 2012a). After attaining maximal contractions with 50 mM K^+^, relaxations were induced with increasing concentrations of the L-type Ca^2+^ channel blocker diltiazem (3^*^10^−9^–3^*^10^−5^ M). To avoid any vasomotor interference due to prostanoids, 10 μM indomethacin was present in all experiments.

### Histology

Segments, which were mounted in the myograph for 7–8 h, were *in-situ* fixed with 4% paraformaldehyde for 24 h, dehydrated and embedded in paraffin. Histological analysis was performed on serial cross sections (5 μm) stained with orcein to visualize elastine. The images were acquired with the Universal Grab 6.1. (IDL) software (Exelis, Boulder, CO) using an Olympus BX40 microscope (Tokyo, Japan). The relative amount of elastin (%) was determined by calculating the number of elastin pixels vs. total number of wall pixels (ImageJ).

### Data presentation and statistical analysis

All results are expressed as mean ± sem with n representing the number of mice. Concentration-response curves were fitted with sigmoidal concentration-response equations with variable slope (GraphPad Prism), which revealed maximal responses (E_max_) and the logarithm of the concentration resulting in 50% of the maximal excitatory or inhibitory effect (EC_50_ or IC_50_) for each vessel segment. Data of the different vessels were compared by One-way or Two-way ANOVA with Bonferroni multiple comparison post-test (GraphPad Prism). A 5% level of significance was selected.

## Results

### Morphological and functional analysis of elastic and muscular arteries

**Figure 1:**
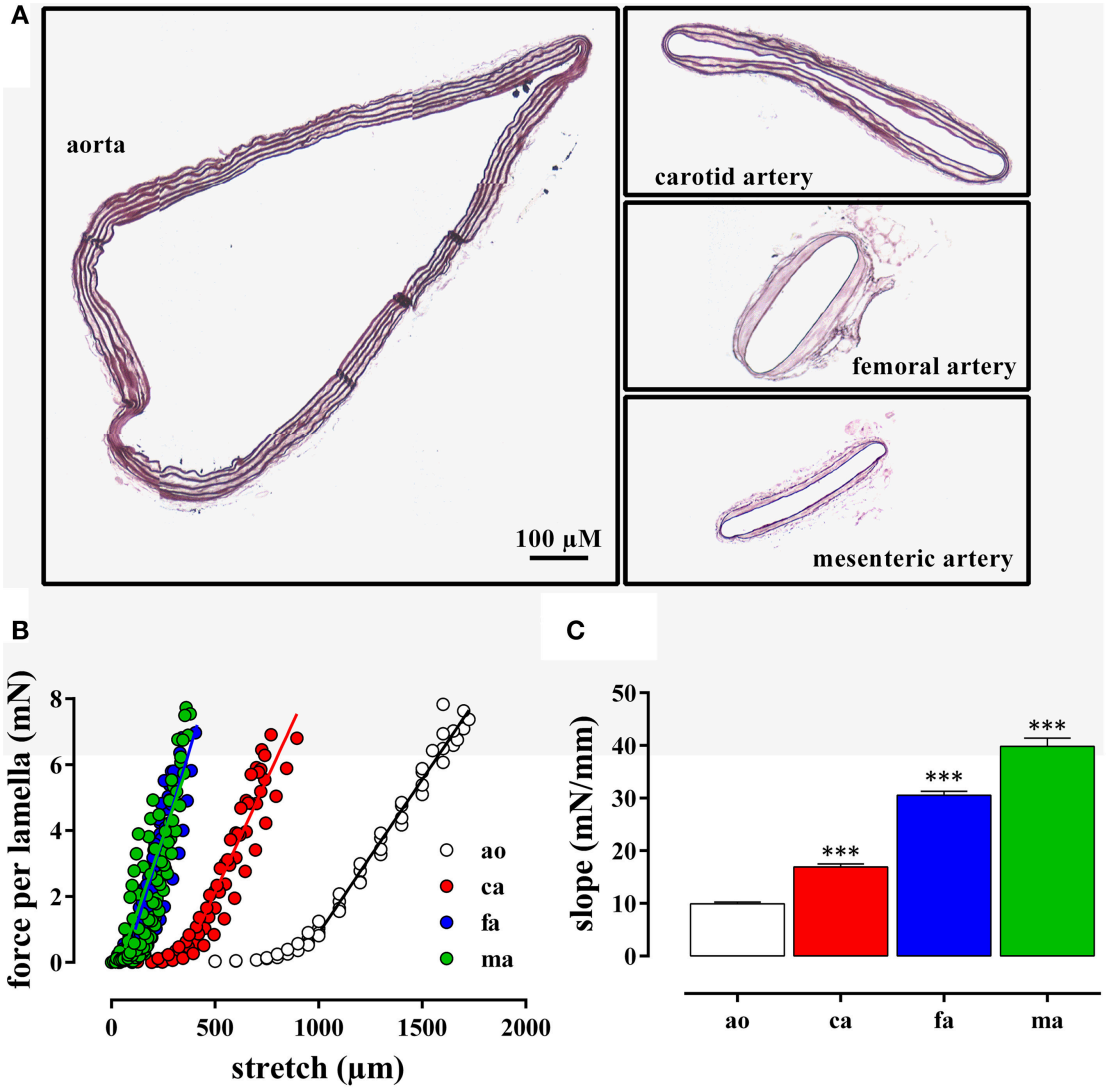
**(A)** Transverse sections through segments of the aorta, the carotid artery, the mesenteric artery, and the femoral artery from the same mouse. The segments were mounted in the myograph for 8 h and transverse sections were stained with orcein to show the elastin layers in the different blood vessels. **(B)** Force per lamella as a function of stretch for the aorta (ao, *n* = 4), the carotid artery (ca, *n* = 6), the femoral artery (fa, *n* = 8), and the mesenteric artery (ma, *n* = 8). **(C)** Slope of the linear part of the different force-stretch relationships shown in **(B)**. All slopes were significantly (^***^P < 0.001) different from each other.

**Figure 2:**
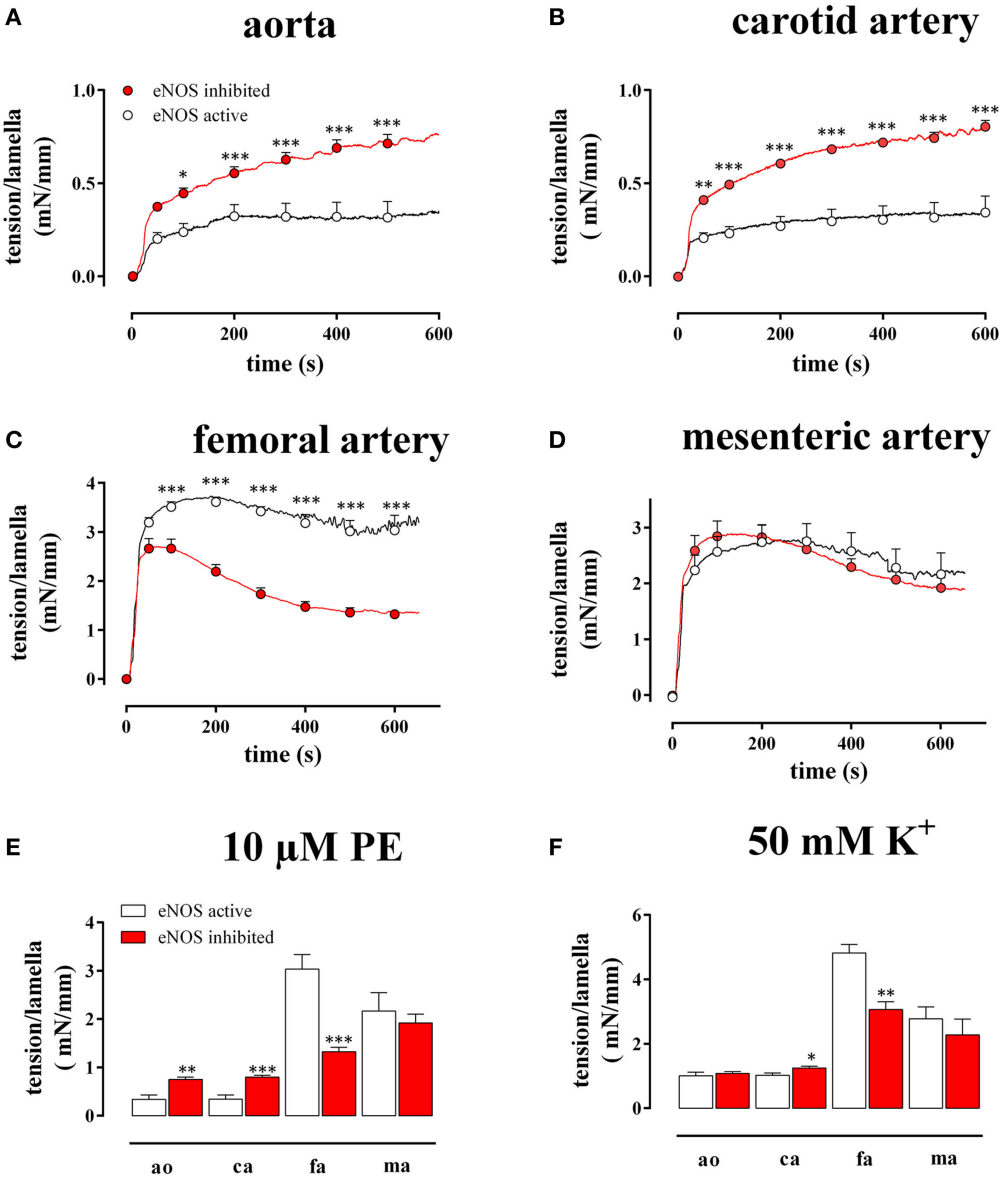
**Tension per lamella as a function of time after addition of 10 μM PE in the absence (eNOS active, open circles) and presence (eNOS inhibited, red) of 300 μM L-NAME in the aorta (A), the carotid artery (B), the femoral artery (C), and the mesenteric artery (D)**. The maximal tension/lamella of aorta (ao), carotid artery (ca), femoral artery (fa), and mesenteric artery (ma) after 600 s tension development by 10 μM PE **(E)** or 50 mM K^+^ **(F)** was calculated. White, eNOS active; red, eNOS inhibited; ^*^, ^**^, ^***^P < 0.05, 0.01, 0.001 eNOS inhibited vs. eNOS active.

The internal circumference extrapolated to 100 mm Hg increased from about 700/750 μm for femoral and mesenteric artery to 1550 for carotid artery and 3230 μm for aorta (Table 1). The amount of elastin per surface area (Figure 1A) was significantly larger (*p* < 0.001, *n* = 4) in the elastic aorta (51.3 ± 1.9%) and carotid artery (47.8 ± 0.9%) as compared to the muscular mesenteric (14.4 ± 1.4%) and femoral arteries (11.8 ± 1.4%), which is compatible with the elastic, respectively, muscular nature of the vessels. Similarly, the number of lamellae, which are the concentric cylindrical building blocks of the arterial wall (Wagenseil et al., 2009), decreased from 4 in the aorta to 2 in the carotid and 1 in the femoral and mesenteric artery. Passive stretch of the arteries elevated force per lamella more gradually in the elastic than the muscular arteries. In the latter, a small increase in diameter (stretch) resulted in a substantial increase of force per lamella (Figure 1B). Thereby, the slope of the force per lamella-stretch relationship (Figure 1C) increased from 10 to 40 mN/mm for the aorta to the mesenteric artery. The muscular nature of the femoral and mesenteric artery was further confirmed by determining active tension per lamella (force per mm) evoked with 10 μM PE or 50 mM K^+^. The tension per lamella was significantly higher in the muscular arteries than in the carotid artery or the aorta (Figures 2E,F). The degree of muscularity decreased from mesenteric ≥ femoral artery > carotid artery > aorta.

**Table 1:** **The internal circumference at 100 mm Hg and slope of the force per lamella vs. stretch relationship for aorta (***n*** = 4), carotid artery (***n*** = 6), femoral artery (***n*** = 8), and first order mesenteric artery (***n*** = 8)**.

**Artery**	**Internal circumference (μm)**	**Slope (force per lamella vs. stretch) (mN/mm)**
Aorta	3230 ± 70 μm (###, $$$, &&&)	9.9 ± 3.4 (##, $$$, &&&)
Carotid artery	1540 ± 50 μm (^***^, $$$, &&&)	16.9 ± 0.6 (^**^, $$$, &&&)
Femoral artery	710 ± 20 μm (^***^, ###)	30.6 ± 0.8 (^***^, ###, &&&)
Mesenteric artery	750 ± 50 μm (^***^, ###)	39.8 ± 1.6 (^***^, ###, $$$)

**, *** P < 0.01, 0.001 vs. aorta;

*##, ###P < 0.01, 0.0001 vs. carotid artery; $$$ P < 0.001 vs. femoral artery; &&& P < 0.001 vs. mesenteric artery*.

### Analysis of basal NO release in mouse arteries

As shown in Figure 2, both in the absence (L-NAME) and in the presence of basal NO release (control), 10 μM PE induced a different time-dependent tension increase in the elastic (Figures 2A,B) compared to the muscular (Figures 2C,D) vessels. Whereas in the elastic arteries, the tension gradually increased for more than 10 min, the tension in the muscular arteries typically rose to a maximum at 50–150 s, then slightly decreased at further time intervals and reached “steady-state” at 10 min. Figure 2E summarizes the “near” steady-state tensions (at 600 s) for the different vessel segments. After inhibition of NO production with 300 μM L-NAME, the PE-induced tension per lamella increased in the aorta by 218 ± 53% (*n* = 4, *P* < 0.001) and in the carotid artery by 276 ± 67% (*n* = 6, *P* < 0.001). In the femoral artery, tension per lamella significantly declined to 78 ± 5% (*n* = 6, *P* < 0.001), whereas tension was not affected in the mesenteric artery segments (105 ± 6%, *n* = 5, *P* > 0.05). When segments were depolarized with 50 mM K^+^ (Figure 2F), basal NO release inhibition increased the maximal tension per lamella only in the carotid artery to 124 ± 7% (*n* = 6, *P* < 0.05). In the aorta (110 ± 8%, *n* = 4) and in the mesenteric artery (81 ± 4%, *n* = 5) it was not significantly affected whereas in the femoral artery segments it significantly decreased to 78 ± 7% (*n* = 6, *P* < 0.01). When both contractile stimuli were compared, basal NO inhibited the maximal contraction of aortic and carotid artery segments significantly (*P* < 0.001) more for PE–than for depolarization-induced contractions (Figures 2E,F).

### Window contraction curves evoked by depolarization

**Figure 3:**
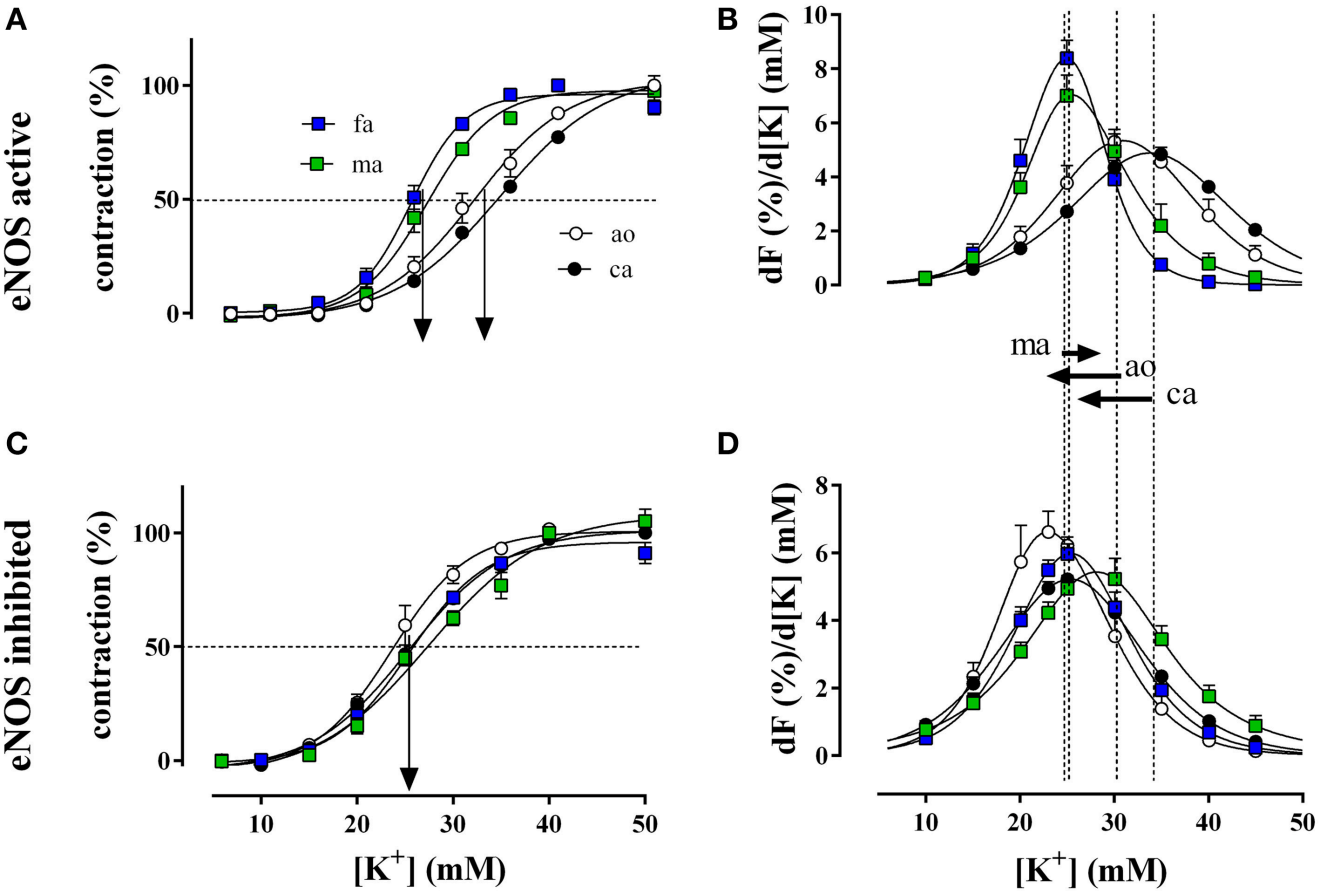
**Relative isometric contractions of the aorta (ao, ***n*** = 4), the carotid artery (ca, ***n*** = 6), the femoral artery (fa, ***n*** = 6), and the mesenteric artery (ma, ***n*** = 5) by increasing K^+^ concentrations (depolarization) in the presence and absence of basal NO (A,C, respectively)**. Basal NO release was inhibited by incubation with 300 μM L-NAME. The first derivative of the individual concentration-response curves of **(A,C)** are displayed in **(B,D)** and correlate with window contraction curves because of L-type Ca^2+^ influx (Fransen et al., 2012a,b). The dashed lines correspond with the EC_50_ of K^+^ or the maximal change of relative tension per concentration of K^+^ with eNOS active, whereas the arrows between **(B)** and **(D)** indicate the shifts of the concentration-contraction curves after inhibition of eNOS with L-NAME for aorta (ao), carotid artery (ca), and mesenteric artery (ma). A shift was absent in fa.

K^+^ concentration-contraction and window contraction curves of segments of the different blood vessels before and after inhibition of eNOS with L-NAME are illustrated in Figure 3. With eNOS active, isometric contractions per lamella at 50 mM K^+^ were 1.01 ± 0.11 mN/mm for the aorta, 1.02 ± 0.07 mN/mm for the carotid artery, 4.98 ± 0.26 mN/mm for the femoral artery, and 3.03 ± 0.40 mN/mm for the mesenteric artery. Blocking eNOS with L-NAME increased the contraction in the elastic arteries to 1.08 ± 0.06 mN/mm (*p* > 0.05) in the aorta and to 1.25 ± 0.06 mN/mm (*p* < 0.05) in the carotid artery and decreased the contractions in the muscular arteries to 3.42 ± 0.41 mN/mm for the femoral artery and to 2.28 ± 0.49 mN/mm for the mesenteric artery.

Relative force development at different K^+^ concentrations (Figure 3A) and the respective window contraction curves (Figure 3B) revealed that in the presence of basal NO release (absence of L-NAME), elastic arteries (aorta and carotid artery) displayed a lower sensitivity to depolarization than muscular arteries (femoral and mesenteric artery). Their window contraction curves were significantly shifted to the right with respect to the curves of the muscular arteries. Inhibition of basal NO release with L-NAME shifted the K^+^ concentration-contraction (Figure 3C) and window contraction (Figure 3D) curves to lower K^+^ concentrations in the elastic arteries only.

The respective half maximal effective K^+^ concentrations in the different conditions and for the different arterial segments are indicated in Table 2. Inhibition of basal NO release with L-NAME only decreased the EC_50_ for K^+^ of the elastic arteries, whereas the EC_50_ of the muscular arteries was not affected.

**Table 2:** **EC_50_-values of K^+^ (mM) in aorta (***n*** = 4), carotid artery (***n*** = 6), femoral artery (***n*** = 6), and mesenteric artery (***n*** = 5) segments in the presence and absence of basal NO release (inhibited with 300 μM L-NAME)**.

**Artery**	**eNOS active**		**eNOS inhibited**	
Aorta	31.3 ± 1.4		23.9 ± 1.1	###
Carotid artery	33.9 ± 0.6		25.4 ± 0.6	###
Femoral artery	24.5 ± 0.7	^***^, $$$	25.3 ± 0.6	
Mesenteric artery	26.5 ± 1.0	^*^, $$$	28.0 ± 0.6	^**^

*, **, *** P < 0.05, 0.01, 0.001 vs. aorta;

*$$$P < 0.001 vs. carotid artery; ###P < 0.001 eNOS inhibited vs. eNOS active*.

### Mouse artery relaxation properties induced by the L-type Ca^2+^ channel blocker diltiazem

**Figure 4:**
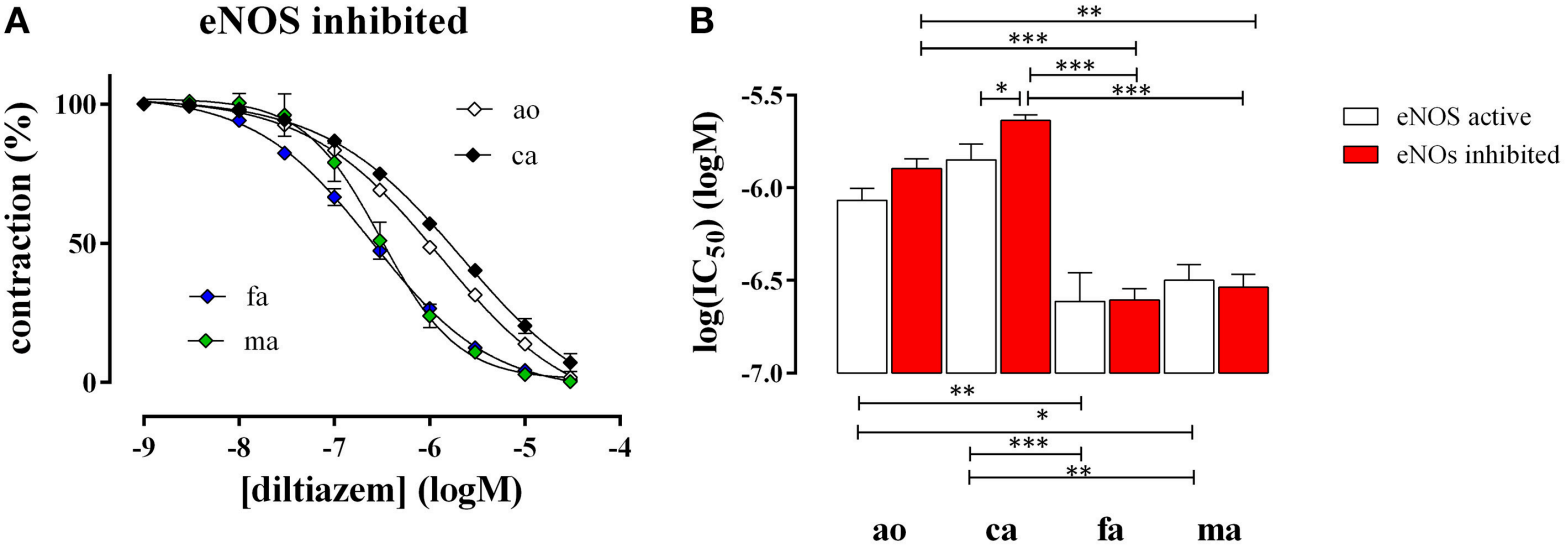
**Relaxation of 50 mM K^+^-elicited contractions by the VGCC blocker diltiazem in aorta (ao, ***n*** = 4), carotid artery (ca, ***n*** = 5), femoral artery (fa, ***n*** = 6), and mesenteric artery (ma, ***n*** = 5). (A)**. Diltiazem concentration-relaxation curves in the presence of 300 μM L-NAME to inhibit basal NO release **(B)** Log(IC_50_) of diltiazem for inhibition of the isometric contractions induced by 50 mM K^+^ in the absence (eNOS active, white) and presence (eNOS inhibited, red) of 300 μM L-NAME to block basal NO release. ^*^, ^**^, ^***^P < 0.05, 0.01, 0.001.

After attaining maximal contraction with 50 mM K^+^, relaxation of the vessel segments was elicited by adding increasing concentrations of the L-type Ca^2+^ channel blocker diltiazem (1^*^10^−9^ to 3^*^10^−5^ M). In all blood vessels diltiazem caused maximal relaxation indicating that the contraction by high K^+^ was completely due to L-type Ca^2+^ channel mediated Ca^2+^ influx, as we have shown before for the aorta (Fransen et al., 2012b). Figure 4 shows the diltiazem concentration-relaxation curves in the presence of the eNOS inhibitor L-NAME (Figure 4A) and the respective log(IC_50_) values for diltiazem in the absence and presence of L-NAME (Figure 4B). Segments of the femoral and the mesenteric artery were significantly more sensitive to diltiazem than segments of the aorta and the carotid artery. In the presence of basal NO release (absence of L-NAME) the affinity for diltiazem increased non-significantly (*p* = 0.08) in the aorta and significantly in the carotid artery, but not in the femoral or the mesenteric artery (Figure 4B).

## Discussion

In this study, we determined the properties of elastic and muscular arteries that may contribute to differential regulation of arterial compliance along the vascular tree. Isometric contractions elicited by α_1_-adrenoceptor stimulation and by depolarizing the membrane potential differed between the elastic arteries (carotid artery and aorta) and the muscular arteries (femoral and mesenteric artery). We were able to show that elastic arteries produced more basal NO, which shifted the window contraction curves to higher K^+^ concentrations and were less sensitive to the L-type Ca^2+^ channel blocker diltiazem than muscular arteries.

### Elastic vs. muscular arteries: passive and active mechanics

Arterial walls are complex tissues composed of different cell populations and extracellular matrix, capable of structural and functional changes in response to direct injury, atherogenic factors, or long-term hemodynamic alterations (Safar, 2010; Safar et al., 2011). The arterial media contains concentric cylindrical lamellae, which serve as building blocks of the vessel wall (Wagenseil et al., 2009). These include two fenestrated sheets of elastin, separated by VSMC, collagen, and extracellular matrix. In mice, the arterial wall of the aorta and carotid artery was built up by 4, respectively, 2 lamellar units, whereas the femoral and mesenteric artery walls contained only 1 lamellar unit. Elastin and collagen are the principal components to determine the passive mechanical properties of the aortic wall. Per unit surface area, the carotid artery and aorta contained more elastin than the femoral and mesenteric artery confirming the elastic nature of the former arteries, and the muscular nature of the latter. As a consequence, stretching the segments of elastic arteries elicited less force per lamella than stretching muscular arteries and α_1_-adrenoreceptor stimulation with PE or VGCC opening with high K^+^ caused higher active force per unit surface in the muscular than in the elastic arteries. In line with these observations are the major physiological functions of elastic and muscular arteries, respectively, pulse-smoothening and blood distribution (Safar, 2010).

### Elastic vs. muscular arteries: basal NO release

A prominent finding of this study is that only elastic arteries released basal NO with important consequences for basal tonus and Ca^2+^ influx via L-type Ca^2+^ channels. Previously, it was already demonstrated that the femoral artery exerted more force in response to the α_1_ adrenergic agonist phenylephrine or to K^+^ elevation as compared to the carotid artery and it was assumed that this was due to PE-induced stimulation of α-adrenoreceptors on endothelial cells in the carotid artery (Crauwels et al., 2000). In aortic segments, however, the high basal output of NO is also present in the absence of α-adrenoreceptor stimulation (Fransen et al., 2012b; van Langen et al., 2012). Therefore, we suggest that even in non-stimulated conditions the elastic, but not muscular arteries, produce large amounts of basal NO. This basal NO attenuates PE-induced contractions significantly and causes significant shifts of the voltage-dependent window contraction curves, especially at moderate depolarizations (Fransen et al., 2012b). At 50 mM K^+^, however, contraction is not affected by NO and the high basal NO release in elastic arteries is masked (Fransen et al., 2012b). The role of basal NO release in the elastic vs. muscular arteries was also evident from the shifts of the window contraction curves. In aorta, inhibition of basal NO release with L-NAME sensitized the window contraction curves to external K^+^ (depolarization; Fransen et al., 2012b). This shift to lower external K^+^ also occurred in the carotid artery, but not in the muscular arteries, confirming the results obtained with α_1_-adrenoceptor stimulation with PE. Because of discrepancies between basal and stimulated NO release, one can speculate about the physiological relevance of the high basal release of NO in elastic arteries. We hypothesize that a major function of this high basal release of NO in the elastic arteries is to avoid stiffening of the arteries in order to maintain their blood pulse-smoothening properties (Peng et al., 2003). We and others have previously shown in rat and in mouse that inhibition of eNOS with L-NAME or knock-out of eNOS caused hypertension and a significant increase of carotid-femoral pulse wave velocity (Isabelle et al., 2012; Leloup et al., 2014). On the other hand, treatment of old mice with sodium nitrite, as a diet-derived source of NO, caused de-stiffening of large elastic arteries and normalization of aortic pulse wave velocity (Sindler et al., 2014). Moreover, the high basal NO release and the shift of the window contraction curve to higher extracellular K^+^ result in a lower sensitivity of the contractile properties of elastic arteries to small variations of their resting membrane potential as indicated by the K^+^ concentration (membrane potential)-contraction curves. The absence of basal NO activity in muscular arteries, on the other hand, makes contraction of medium to small-sized arteries to vary considerably with small alterations of membrane potential, allowing stringent regulation of arterial diameter and blood flow according to moment-to-moment needs. It should be mentioned that in the femoral artery, both PE-and 50 mM K^+^-induced contractions decreased with basal NO release inhibition with L-NAME. Furthermore, in mesenteric artery, there was a reverse shift of the window contraction curves with L-NAME. These effects, however, also occurred in the absence of L-NAME, were time-dependent (data not shown) and, hence, were not due to changes in NO bioavailability.

### Elastic vs. muscular arteries: sensitivity to diltiazem

In the absence of basal NO production or presence of L-NAME, the sensitivity of all blood vessels to K^+^ was similar. Nevertheless, even then, the muscular arteries were significantly more sensitive to the L-type Ca^2+^ channel inhibitor diltiazem, which has a high affinity for window contractions (Fransen et al., 2012a; Michiels et al., 2014). These results suggest the occurrence of different populations of L-type Ca^2+^ channels in the elastic and the muscular arteries. This is in line with the non-homogeneous L-type Ca^2+^ channel population distribution in the cardiovascular system. Thereby, some isoforms display hyperpolarized window currents and enhanced state-dependent block by nifedipine (Liao et al., 2004, 2007; Zhang et al., 2010).

In conclusion, muscular arteries such as femoral and mesenteric arteries and elastic arteries such as the aorta and carotid artery differ in their passive and active contractile properties. To the best of our knowledge, this is the first study to report that elastic arteries display significantly higher basal NO efficacy and large shifts of the window contraction curves to depolarized potentials as compared to muscular arteries. In the absence of basal NO, voltage-dependent VGCC-mediated contractions were similar in elastic and muscular arteries. However, the K^+^-induced contraction was inhibited by the VGCC channel blocker diltiazem with significantly higher sensitivity in muscular arteries, suggestive for different VGCC isoform populations in both vessel types. We speculate that the observed differences in basal NO production in elastic and muscular arteries contribute to the differential effects of aging on both vessel types. This is supported by studies showing significant improvement of arterial compliance after treatment with enhancers of endothelial function (Sindler et al., 2011; Santos-Parker et al., 2014). The major limitations of this study are the lack of direct measurement of elasticity, basal NO production, and VGCC isoforms in elastic and muscular arteries. Earlier studies both in mice and humans already demonstrated different elastic and muscular artery stiffness. On the other hand, assessment of basal NO production is technically difficult due to the low NO concentrations and the different spliced variants of the calcium channel have not yet been studied in detail, and certainly not in different vascular beds, hampering PCR-based VGCC isoform determination. Addressing these challenges during future research will contribute to directly demonstrate the relationship between basal NO production, VGCC isoforms, and arterial stiffness.

## Author contributions

AL, design of research, performed experiments, analyzed data, interpreted results of experiments, drafted manuscript, prepared figures, approved final version of manuscript and accountability for accuracy and integrity of the work. CV, design of research; analyzed data; interpreted results of experiments, reviewed and revised manuscript; approved final version of manuscript and accountability for accuracy and integrity of the work. AH, performed experiments, interpreted results of experiments, revised manuscript, approved final version of manuscript and accountability for accuracy and integrity of the work. DS, interpreted results of experiments, edited and revised manuscript, approved final version of manuscript and accountability for accuracy and integrity of the work. GD, interpreted results of experiments, edited and revised manuscript, approved final version of manuscript and accountability for accuracy and integrity of the work. PF, design and conception of research, edited and drafted manuscript, analyzed and interpreted data of experiments, prepared figures, revised manuscript, approved final version of manuscript and accountability for accuracy and integrity of the work.

### Conflict of interest statement

The authors declare that the research was conducted in the absence of any commercial or financial relationships that could be construed as a potential conflict of interest.

## Abbreviations

eNOS: Endothelial nitric oxide synthase
L-NAME: NΩw-nitro-L-arginine methyl ester
NO: Nitric oxide
PE: Phenylephrine
VGCC: Voltage-gated Ca^2+^ channel
VSMC: Vascular smooth muscle cell.
